# Isolated biomolecules of pharmacological interest in hemostasis from Cerastes cerastes venom

**DOI:** 10.1186/1678-9199-19-11

**Published:** 2013-05-01

**Authors:** Fatah Chérifi, Fatima Laraba-Djebari

**Affiliations:** 1 USTHB, Faculty of Biological Sciences, Laboratory of Cellular and Molecular Biology, BP 32 El-Alia, Bab Ezzouar, Algiers, Algeria; 2 USTHB, Faculty of Biological Sciences, University of Sciences and Technology Houari Boumedienne, BP 32 El Alia, Bab Ezzouar, Algiers, Algeria

**Keywords:** *Cerastes cerastes* venom, Proteinases, Phospholipases A2, Platelets, Blood-clotting, Hemostasis

## Abstract

Biomolecules from *Cerastes cerastes* venom have been purified and characterized. Two phospholipases isolated from *Cerastes cerastes* venom share 51% of homology. CC2-PLA2 exhibits antiplatelet activity that blocks coagulation. CCSV-MPase, a non-hemorrhagic Zn^2+^-metalloproteinase, significantly reduced the plasmatic fibrinogen level and hydrolyzes only its Bβ chain. Serine proteinases such as RP34, afaâcytin and CC3-SPase hydrolyze the fibrinogen and are respectively α, αβ and αβ fibrinogenases. In deficient human plasma, afaâcytin replaces the missing factors VIII and IX, and activates purified human factor X into factor Xa. It releases serotonin from platelets and directly aggregates human (but not rabbit) blood platelets. RP34 proteinase also had no effect on both human and rabbit blood platelet aggregation. CC3-SPase revealed a pro-coagulant activity. However, the insolubility of the obtained clot indicates that CC3-SPase does not activate factor XIII. In addition, CC3-SPase clotting activity was carried out with human plasmas from volunteer patients deficient in clotting factors. Results showed that CC3-SPase shortens clotting time of plasma deficient in factors II and VII but with weaker clotting than normal plasma. The clotting time of plasma deficient in factor II is similar to that obtained with normal plasma; suggesting that CC3-SPase is able to replace both factors IIa and VII in the coagulation cascade and thus could be involved in the blood clotting process via an extrinsic pathway. These results imply that CC3-SPase and afaâcytin could repair hemostatic abnormalities and may replace some factors missing in pathological deficiency. Afaâcytin also exhibits α fibrinase property similar to a plasmin-like proteinase. Despite its thrombin-like characteristics, afaâcytin is not inhibited by plasmatic thrombin inhibitors. The procoagulant properties of afaâcytin might have potential clinical applications.

## Introduction

Serine proteases and phospholipases A2 isolated from snake venoms act on the hemostatic system as procoagulants, anticoagulants, pro- or anti-platelet aggregants. Some of these isolated molecules, mainly from *Viperidae* venoms, are used in diagnosis or treatment of thrombotic diseases and ischemic heart disease. Metalloproteinases can cause hemorrhage after accidental or experimental envenomation. However, some of these metalloproteinases are directly involved in the clotting of blood as they can act on fibrinogen and/or fibrin; they are called in this case fibrino(geno)lytic metalloproteinases. Their fibrinolytic activity makes them potent inhibitors of blood coagulation. Phospholipase A2 exert their anticoagulant effect by their ability to inhibit platelet aggregation due to their high affinity to bind to activated factor Stewart (FXa). All these biological effects based on their direct involvement in hemostasis, let consider these molecules as potential tools or biomarkers in blood diseases.

## Review

Viperidae and Crotalidae venoms are rich sources of hydrolytic enzymes and produce a complex pattern of clinical and toxic effects such as coagulation disorders, hemorrhage and necrosis [1-10]. Some of venom components act at various stages of the coagulation cascade. These components perform antagonistic functions, whilst some of them act synergistically. Therefore, the venom toxicity cannot be attributed to only one component [11]. However, most venom components produce beneficial effects when they act alone [12]. Snake venom also contains non-protein components including citrate, metal ions, carbohydrates, nucleotides as well as low concentrations of free amino acids and lipids [13-15].

Phospholipases A2 (PLA2s) represent more than 10% of the dry weight of the snake venoms from which they are isolated. PLA2s isolated from Viperidae venoms consist of 125–130 amino acid residues cross-linked by seven disulfide bonds which confer stability on the molecule and are calcium-dependant [16]. In addition to their hydrolytic activity, PLA2s may display many other activities, such as edematous, neurotoxic, cardiotoxic, hemolytic, convulsive, antiplatelet, antitumoral and anticoagulant properties. Based on their anticoagulant activity, PLA2s can be clinically useful against thrombotic diseases and for the diagnosis and treatment of hemostatic disorders [17]. The anticoagulant activity is due to their ability to inhibit platelet aggregation through factor Xa (FXa) blockade. Based on their direct involvement in the hemostatic cascade, PLA2 could also be used as tools or biomarkers in blood diseases.

Metalloproteinases found in Viperidae venoms may cause local hemorrhaging following accidental or experimental intradermal or subcutaneous injection of venom [18]. Some of them are known to display fibrino (geno)lytic activity. Fibrino(geno)lytic metalloproteinases dissolve fibrin clots and prevent clot formation by hydrolyzing fibrinogen, thus enhancing the toxic effect of hemorrhagic metalloproteinases, giving rise to pathological bleeding [19]. However, similar to PLA2s, metalloproteinases could be clinically useful against thrombotic diseases thanks to their potential use in laboratory tests or as therapeutic agents [20,21]. These proteinases may be useful for investigating the mechanisms of blood coagulation and platelet aggregation [11,12].

In addition to their beneficial effects, venom molecules are the cause of health problems after snake envenomation. Annually, more than 100,000 deaths are recorded worldwide, including 20,000 on African continent, while 400,000 victims retain severe and permanent functional sequelae [3]. Epidemiological data estimate envenomation cases at more than 5 million per year, with a mortality rate of 2.5%. In tropical Africa, Viperidae bites are responsible for 90% of envenomations [22]. Currently, the only available specific treatment against viper envenomation is immunotherapy [23-26]. Indications for this therapy, its mode of administration and type of antibody preparation in the form of F (ab’)_2_ or Fab still need to be elucidated [27-30]. Improvement of this treatment requires knowledge of the kinetic distribution of venom in vascular and tissue compartments.

*Cerastes cerastes* venom is a mixture of various proteins with broad biological and physiological activities; most of them are proteinases while some have been well characterized. Most of these proteins act on blood coagulation, including PLA2, the thrombin-like enzymes RP34 and afaâcytin, anticoagulant protease fraction, aggregant serine protease, hemorrhagic metalloproteinase CcH1, and the non-hemorrhagic metalloproteinase CCSV-MPase [11,12,31-35]. In this paper, we report pharmacological activity of biomolecules isolated from *Cerastes cerastes* on hemostasis process.

### Biochemical properties of biomolecules and their proteomic identification

Several molecules from *Cerastes cerastes* venom act on hemostasis, such as RP34, a serine proteinase which consists of two subunits of 48.5 kDa [32]. Another serine proteinase, a thrombin-like molecule denominated afaâcytin, was purified and characterized [11]. Afaâcytin presents caseinolytic, arginine-esterase and amidase activities. It is a homodimeric proteinase with two subunits, alpha and beta, which have the same apparent molecular mass (40.0 kDa for each unit) and are indistinguishable in the absence of reduction or/and deglycosylation [11]. Both α and β chains are N-glycosylated. The two chains present the same N-terminal sequence (20 residues) which is similar to the sequence of other proteinases isolated from snake venom.

Three molecules – CC2-PLA2, CCSV-MPase and CC3-SPase – were characterized by proteomic analysis. Results showed some sequence similarity with other homologous enzymes isolated from several venoms [11,12,17,31-34]. CC2-PLA2, another PLA2 found in the same venom, presents 51% sequence homology with a previously purified molecule from the same venom by Laraba-Djebari *et al*. [32] (accession number in NCBInr is gi |129506|), *i*.*e*. 61 out of 120 amino acid residues are common to the two PLA2s [31]. The peptide sequence of the new PLA2 was obtained by alignment with sequences of other venom PLA2s. Some snake venom proteinases were identified sharing sequence homology with CCSV MPase, four of which belonged to the metalloproteinase family (Group III snake venom metalloproteinase and Zn^+2^-metalloproteinase disintegrin) [17]. Three of these proteins corresponded to *Cerastes vipera* venom and the others to venom proteins of different snake species [17].

All of these proteins were identified with at least two unique specific peptides and presented similarities with the purified CCSV-MPase. Previous studies showed that CCSV-MPase, characterized by SDS-PAGE analysis, could be classified as a member of the high-molecular-mass metalloproteinase family, due to its molecular mass estimated at 70 kDa in both reducing and non-reducing electrophoresis conditions [12]. Furthermore, the partial amino acid sequence of CCSV-MPase was identified by MALDI-TOF MS/MS analysis. Based on its molecular mass and partial amino acid sequence, CCSV-MPase may be classified in the P-III class of SVMPs containing a disintegrin-like metalloproteinase, with cystein-rich domains.

It is well established that some venom components have beneficial effects when acting in isolation. *Cerastes cerastes* venom is a mixture of protein components with multiple actions including coagulation [11,32,36,37]. These proteins may induce hemorrhage and capillary permeability disorders, through their disintegrin domain or related proteins that disrupt primary hemostasis by acting on platelet adhesion. Thus, a single molecule can be endowed with several activities. The structural differences between proteins, natural factors of hemostasis, as well as the multiplicity of target components of the same venom, are elements that could explain the efficiency of partial immunotherapy [15]. Fibrinogenases (serine proteinases or metalloproteinases) are widespread in Viperidae venoms. They hydrolyze fibrinogen and/or degrade the fibrin clot, enhancing the effect of hemorrhagic metalloproteinases that give rise to pathological bleeding.

### Coagulant and fibrinogenolytic activities of isolated molecules

Fibrinogen is a glycoprotein of 340 kDa with three polypeptide chains; Aα (67 kDa), Bβ (50 kDa) and γ (43 kDa) linked by disulfide bonds. It can be hydrolyzed by thrombin, thus producing fibrin components and fibrinopeptides. Thrombin activity (control) on fibrinogen demonstrated the release of fibrinopeptide A (FpA) followed by fibrinopeptide B (FpB).

Proteolytic enzymes of *Cerastes cerastes* venom were identified as α, β or γ fibrinogenases depending on their ability to hydrolyze the fibrinogen *in vitro*. SDS-PAGE analysis of fibrinogen in the presence of venom revealed two entities (55 kDa and 50 kDa) indicating activities of α- and β-fibrinogenase. Purification and characterization of three procoagulant proteinases (RP34, afaâcytin and CC3-SPase proteinase) showed fibrinogenolytic activities when analyzed by SDS-PAGE, afaâcytin and RP34 displayed, respectively, α,ß-fibrinogenase and α-fibrinogenase activity [11,32,34]. Like afaâcytin, CC3-SPase is also characterized as an α,β-fibrinogenase due to the release of both A and B fibrinopeptides.

Susceptibility of afaâcytin to diisopropyl fluorophosphate and benzamidine indicates the presence of a serine and an aspartic (or glutamic) acid residues in the catalytic site. Calcium is required for structural cohesion of the afaâcytin molecule [11]. CCSV-MPase cleaves only the Bβ chain of fibrinogen and exerted no action on Aα or γ chains. This property contrasts with those of other SVMPs which preferentially cleave only the Aα-fibrinogen chain. However, these metalloproteinases, belonging to the PI class of SVMPs, present low molecular mass, with only the metalloproteinase domain, as in the case of fibrolase purified from *Akgistrodon contortrix contortrix*, piscivorase II of *Akgistrodon piscivorus piscivorus*, lebetase purified from the venom of *Vipera lebetina*, neuwiedase from *Bothrops neuwiedi* venom, the atroxase of *Crotalus atrox* and leucurolysins from venom of *Bothrops leucurus*[5,38-42].

Proteinases (afaâcytin, RP34, CC3-SPase and CCSV-MPase) showed caseinolytic activity as crude venom. CC3-SPase displayed arginine ester hydrolase activity while the CCSV-MPase does not. Both molecules presented a high amidolytic activity similar to that of crude venom. Previous results revealed that the use of specific inhibitors for serine proteinases and metalloproteinases showed that CC3-SPase is a thrombin-like Ca^2+^-dependent serine proteinase. Afaâcytin isolated from the venom of *Cerastes cerastes* showed that Ca^2+^ is essential for its activity not only as a cofactor but can contribute to the stability or structural cohesion of the enzyme [11]. CCSV-MPase appears to be a zinc-dependent metalloproteinase given that metal chelators, EDTA and 1,10-phenanthroline completely inhibited its proteolytic activity, which also suggested that unlike CC3-SPase, Ca^2+^ is not required for its catalytic activity. The sensitivity of the serine proteinase CC3-SPase, a specific inhibitor of thrombin (heparin and antithrombin III) may indicate that the receptor of CC3-SPase is identical to that of thrombin. Given its procoagulant properties and insensitivity to thrombin-specific plasma inhibitors, afaâcytin might be interesting to employ as a hemostatic agent in some types of hemorrhage, such as post-operative thrombocytopenia [11].

Serine-proteinases hydrolyze fibrinogen by acting on the two chains, α and β, of this substrate thereby causing the formation of a fragile fibrin clot. CCSV-MPase acts on the β chain of fibrinogen resulting in the release of only fibrinopeptide B. Afaâcytin, as a component of the venom (2% w/w), hydrolyzes fibrinogen in the same manner that CC3-SPase degrades firstly the Aα chain and then, 24 hours later, the Bβ chain, leading to a fragile clot, which suggests that CC3-SPase, similarly to afaâcytin, is unable to activate the factor XIII responsible for the resistance of the fibrin clot [11,34]. CCSV-MPase cleaves only the Bβ chain of fibrinogen and exerts no activity on Aα and γ chains. CCSV-MPase properties may allow its use as a therapeutic agent in some pathologies that require anticoagulant administration.

Most thrombin-like enzymes (TLE) isolated from snake venoms act on fibrinogen by hydrolyzing one chain rather than two, although the cleavage site is the same (Arg16-Gly17) as the α chain (Arg15- Gly16) on β chain, by releasing fibrinopeptides A or B as CCSV-MPase which degrades only the β chain of fibrinogen. CC3-SPase shortened the clotting time of plasma deficient in factor VII and II with a weaker clot than that formed with normal plasma. The clotting time of plasma deficient in factor II is similar to that obtained with normal plasma after the action of serine protease CC3-SPase, which suggests that this molecule is able to replace factors IIa and VII.

Procoagulant and anticoagulant snake venom components often act at later stages of the coagulation cascade. The main targets of these components are fibrinogen, prothrombin, factor X and platelets [3,5,11,12,17].

Several molecules have been purified from Viperidae and Crotalidae venoms and characterized as FX activator factors that are used as biomarkers in many hemostatic disorders. Indeed, thrombin-like components serve as structural models to extend our understanding of the structure-function relationships of blood coagulation factors, some of which have been clinically used for the treatment of thrombotic diseases, and are employed as tools in clinical assays [43].

Combination of gel filtration and ion-exchange chromatography proved to be successful in obtaining milligram quantities of new pure TLEs from the venoms of *Crotalus durissus terrificus* and *Crotalus durissus collilineatus*[43]. Functional characterization indicates that both enzymes preferentially degrade the Bβ chain of bovine fibrinogen and present edema-inducing and coagulant activities. However, the TLE from *Crotalus durissus collilineatus* venom showed twofold higher coagulant activity with a minimum coagulant dose (MCD) of 0.6 μg/μL, whereas the enzyme isolated from *Crotalus durissus terrificus* indicated an MCD of 1.5 μg/μL [43].

Recently, a TLE denominated BpSP-I was isolated from *Bothrops pauloensis* snake venom; its biochemical, enzymatic and pharmacological characteristics were determined. BpSP-I showed high clotting activity upon bovine and human plasma and was inhibited by PMSF, benzamidine and leupeptin. Moreover, this enzyme showed stability when examined at different temperatures (−70 to 37°C), pH values [3-9] or in the presence of divalent metal ions (Ca^2+^, Mg^2+^, Zn^2+^ and Mn^2+^). BpSP-I showed high catalytic activity upon substrates, such as fibrinogen, TAME, S-2238 and S-2288. It also showed kallikrein-like activity, but was unable to act upon factor Xa or plasmin substrates [44].

Reducing blood viscosity is often required in the treatment of thrombotic and ischemic heart diseases. Defibrinogenation of the plasma by some enzymes from snake venoms is of interest. Indeed, all of these defibrinogenating biomolecules sharing these properties could be used as tools in clinical applications and in basic research. Further studies, in pharmacology and toxicology should be undertaken to determine their mode of action *in vivo*.

### Effect of molecules on platelet function

Biological characterization of CCSV-MPase and CC2-PLA2 has been shown to be highly anti-aggregative in relation to human platelets. The antagonistic effect of CC3-SPase is of interest in the context of the antiplatelet action of the hemostatic system, and may be an effective tool for reducing blood viscosity, a property that is often necessary in the treatment of thrombotic diseases and ischemic heart syndrome due to platelet aggregation. Previous studies have already demonstrated that afaâcytin may replace the missing factors VIII and IX in deficient plasmas, and activate purified human factor X into factor Xa [11]. It releases serotonin from platelets and directly aggregates human (but not rabbit) blood platelets. On the other hand, RP 34 has no effect on platelet aggregation [32]. Several anticoagulant PLA2s from snake venoms have been isolated and well characterized. Recently, two phospholipases, known as CC-PLA2-1 and CC-PLA2-2 with antiplatelet aggregation activity, were isolated from *Cerastes cerastes* venom [45]. An anticoagulant PLA2 was isolated and characterized as an inhibitor of the prothrombinase complex through its specific binding to FX [46]. Ammodytoxin A (Atxa) and its natural ammodytoxin isoform C were isolated from *Vipera ammodytes ammodytes* venom and belong to group IIA secreted phosphoplipases. These two isoforms differ only by two amino acid residues (Phe 124 > Ile and Lys128 > Glu), but there are significant differences in toxicity. The mechanism by which they block coagulation has been elucidated. Complementary experiments using surface plasmon resonance showed complete inhibition of binding to FXa through calmodulin (CaM). The crystal structure showed that the C-terminal region required for binding to FXa and CaM is highly exposed and accessible for interaction with receptor proteins in the monomeric and dimeric forms of ammodytoxin [45].

## Conclusion

Viperidae venoms, considered to be one of the most important bioresources, include pharmacologically active molecules such as proteinases (metalloproteinases and serine proteinases) and phospholipase A2 [46,47]. All of these molecules are of interest in biotherapy as biomedicines or may be used as diagnostic tools. Proteases and PLA2 act on the hemostatic system as procoagulants, anticoagulants, and as agents of pro- or anti-platelet aggregation. Some of these molecules, especially those isolated from Viperidae venoms, are used in the diagnosis and treatment of thrombotic and heart diseases. Some components act synergistically at different stages of the coagulation cascade [48].

Constituents of Viperidae venoms contain two categories of components that act antagonistically through activation or inhibition of coagulation factors and platelet aggregation. These compounds, able to hydrolyze the coagulation factors with high specificity, are divided into serine proteinases and metalloproteinases. Phospholipases also display potent inhibition of platelet aggregation. Biomolecules of snake venoms are of great fundamental diagnostic and therapeutic interest. Therapeutically, proteinases from Viperidae venoms are widely used as anticoagulants. Furthermore, they are valuable tools for understanding the different mechanisms of hemostasis and are also used in the diagnosis of dysfunctions related to coagulation factors such as enzyme activity in thrombin-like venoms that are used for the fibrinogenopathy screening. Venoms are also used for diagnostic analysis of various coagulation factors (factors V, VII, X, platelet factor III, protein C and factor of Willbrand). Snake venom proteases are useful tools for studying coagulation reactions.

### Ethics committee approval

The present study was approved by the Ethics Committee.

## Competing interests

The authors declare no conflicts of interest.

## Authors’ contributions

Both authors collaborated in this work; they read and approved the final manuscript. FLD carried out the purification and characterization of Serine proteinases; RP34 and Afaâcytin and drafted the manuscript. FC with contribution of FLD, purified and characterized the molecules (CC2-PLA2, CC3-SPase and CCSV-MPase).

## References

[B1] Rivière G Bon C Immunothérapie antivenimeuse des envenimations ophidiennes: vers une approche rationnelle d’un traitement empirique Ann Inst Pasteur 1999 10 2 173 182 10.1016/S0924-4204(99)80032-8

[B2] Chippaux JP Goyffon M Venoms, antivenoms and immunotherapy Toxicon 1997 36 6 823 846 9663690 10.1016/s0041-0101(97)00160-8

[B3] Braud S Bon C Wisner N Snake venom proteins acting on haemostasis Biochimie 2000 82 9–10 851 859 11086215 10.1016/s0300-9084(00)01178-0

[B4] Abib H Laraba-Djebari F Effect of 60Co gamma radiation on toxicity and haemorrhagic, myonecrotic and edema-forming activities of *Cerastes cerastes* venom Can J Physiol Pharmacol 2003 28 12 1141 1147 10.1139/y03-121 14719031

[B5] Castro HC Zingali RB Albuquerque MG Pujol-Luz M Rodrigues CR Snake venom thrombin-like enzymes: from reptilase to now Cell Mol Life Sci 2004 61 7–8 843 856 15095007 10.1007/s00018-003-3325-z PMC11138602

[B6] Castro HC Rodrigues CR Current status of snake venom thrombin-like enzymes Toxin Rev 2006 25 3 291 381 10.1080/15569540600567321

[B7] Kornalik F Shier WT, Mebs D, Dekker M Toxins affecting blood coagulation and fibrinolysis Handbook of toxicology 1990 New York 697 709

[B8] Liu S Sun MZ Greenway FT A novel plasminogen activator from *Agkistrodon blomhoffi ussurensis* venom (Abusv-Pa): purification and characterization Biochem Biophys Res Commun 2006 384 4 1279 1287 16919241 10.1016/j.bbrc.2006.07.183

[B9] Hamza L Girardi T Castelli S Gargioli C Cannata S Patamia M Isolation and characterization of a myotoxin from the venom of *Macrovipera lebetina transmediterranea* Toxicon 2010 56 3 381 390 10.1016/j.toxicon.2010.04.001 20398688

[B10] Hamza L Gargioli C Castelli S Rufini S Laraba-Djebari F Purification and characterization of a fibrinogenolytic and hemorrhagic metalloproteinase isolated from *Vipera lebetina* venom Biochimie 2010 92 7 797 805 10.1016/j.biochi.2010.02.025 20188789

[B11] Laraba-Djebari F Martin-Eauclaire MF Mauco G Matchot P Afaâcytin and α, β-fibrinogenase from Cerastes cerastes (horned viper) venom, activates purified Factor X and induces serotonin release from human blood platelet Eur J Biochem 1995 233 3 756 765 10.1111/j.1432-1033.1995.756_3.x 8521839

[B12] Chérifi F Rousselle JC Namane A Laraba-Djebari F Zn2+: a required ion for procoagulant metalloproteinase (CCSV-MPase) activities, isolated from Cerastes cerastes Venom. SFET Editions, Toxins and Ion transfers, E-Book RT19 2011 161 164

[B13] Freitas MA Geno PW Summer LW Cooke ME Hudenberg SA Ownby CL Citrate is a major component of snake venoms Toxicon 1992 30 4 461 464 10.1016/0041-0101(92)90542-D 1626327

[B14] Bieber AL Lee CY Metal and non-protein constituents in snake venoms Snake venoms 1979 Berlin: Springer 295 304

[B15] Mion G Olive F Hermandez E Martin YN Viellefosse S Goyffon M Action des venins sur la coagulation sanguine: diagnostic des syndromes hémorragiques Bull Soc Path Exot 2002 95 3 132 138 12404853

[B16] Kini RM Venom phospholipase A2 enzymes: structure, function and mechanism 1997 England: Chichester 1 511

[B17] Chérifi F Rousselle JC Namane A Laraba-Djebari F CCSV-MPase, a novel procoagulant metalloproteinase from *Cerastes cerastes* venom: purification, biochemical characterization and protein identification Protein J 2010 29 466 474 10.1007/s10930-010-9273-1 20717713

[B18] Kamiguti AS Platelets as target of snake venom metalloproteinases Toxicon 2005 45 8 1041 1049 10.1016/j.toxicon.2005.02.026 15922773

[B19] Leonardi A Fox JW Trampus-Bakija A Krizaj I Ammodytase, a metalloproteinase from *Vipera ammodytes ammodytes* venom, possesses strong fibrinolytic activity Toxicon 2007 49 6 833 842 10.1016/j.toxicon.2006.12.003 17250863

[B20] Swenson S Markland FS Jr Snake venom fibrin(ogen)olytic enzymes Toxicon 2005 45 8 1021 1039 10.1016/j.toxicon.2005.02.027 15882884

[B21] Hutton RA Warrel DA Action of snake venom components on the haemostatic system Blood Rev 1993 7 3 840 855 10.1016/0268-960x(93)90004-n 8241832

[B22] Chippaux JP Lang J Amadi-Eddine S Fagot P Le Mener V Short report: treatment of snake envenomations by a new polyvalent antivenom composed for highly purifyied F(ab’)2. Results of a clinical trial in northern Cameroon Am J Trop Med Hyg 1999 61 6 1017 1018 10674688 10.4269/ajtmh.1999.61.1017

[B23] Hawgood B Doctor Albert Calmette 1863–1933: founder of antivenomous serotherapy and of antituberculous BCG vaccination Toxicon 1999 37 9 1241 1258 10.1016/S0041-0101(99)00086-0 10400286

[B24] Krifi MN El AM Dellagi K The improvement and standardization of antivenom production in developing countries: comparing antivenom quality, therapeutical efficiency, and cost J Venom Anim Toxins 1999 5 2 128 141

[B25] Ferreira Junior RS Nascimento N Couto R Alves JB Meira DA Barraviera B Laboratory evaluation of young ovines inoculated with natural or 60Co-irradiated *Crotalus durissus terrificus* venom during hyperimmunization process J Venom Anim Toxins incl Trop Dis 2006 12 4 620 631

[B26] Oussedik-Oumehdi H Laraba-Djebari F Irradiated *Cerastes cerastes* venom as a novel tool for immunotherapy Immunopharmacol Immunotoxicol 2008 30 1 37 52 10.1080/08923970701812324 18306103

[B27] Chippaux JP Goyffon M Venoms, antivenoms and immunotherapy Toxicon 1998 36 6 823 846 10.1016/S0041-0101(97)00160-8 9663690

[B28] Morais VM Massaldi H Snake antivenoms: adverse reactions and production technology J Venom Anim Toxins 2009 15 1 2 18

[B29] Theakston RD Warrell DA Griffiths E Report of a WHO workshop on the standardization and control of antivenoms Toxicon 2003 41 5 541 557 10.1016/S0041-0101(02)00393-8 12676433

[B30] Wilde H Thipkong P Sitprija V Chaiyabutr N Heterologous antisera and antivenins are essential biologicals: perspectives on a worldwide crisis Ann Intern Med 1996 125 3 233 236 10.7326/0003-4819-125-3-199608010-00012 8686982

[B31] Laraba-Djebari F Martin-Eauclaire MF Purification and characterization of a phospholipase A2 from *Cerastes cerastes* (horn viper) snake venom Toxicon 1990 28 6 637 646 10.1016/0041-0101(90)90252-3 2402760

[B32] Laraba-Djebari F Martin-Eauclaire MF Marchot PA A fibrinogen-clotting serine proteinase from *Cerastes cerastes* (horned viper) with arginine - esterase an amidase activities, purification, characterization and kinetic parameter determination Toxicon 1992 30 1 1399 1410 1485336 10.1016/0041-0101(92)90515-7

[B33] Chérifi F Laraba-Djebari F Purification et caractérisation d’une fraction anti-coagulante et protéolytique du venin de Cerastes cerastes. Rencontres en Toxinologie, «Toxines émergentes: nouveaux risques» Lavoisier, Editions TEC et DOC 2007 234 23523681531

[B34] Chérifi F Laraba-Djebari F Mise en évidence et Caractérisation d’une Fraction Coagulante et Agrégante du venin de Cerastes cerastes. Toxines et fonctions cholinergiques neuronales et non neuronales. Editions de la SFET 2008 153 154

[B35] Boukhalfa-Abib H Meksem A Laraba-Djebari F Purification and biochemical characterization of a novel hemorrhagic metalloproteinase from horned viper (*Cerastes cerastes*) venom Comp Biochem Physiol C Toxicol Pharmacol 2009 150 2 285 290 10.1016/j.cbpc.2009.05.008 19470410

[B36] El-Asmar MF Shaban E Hagag M Swelam N Tu A Coagulant component in *Cerastes cerastes* (Egyptian sand viper) venom Toxicon 1986 24 11–12 1037 1044 3564057 10.1016/0041-0101(86)90130-3

[B37] Bazaa A Marrakchi N El Ayeb M Sanz L Calvete JJ Snake venomics: comparative analysis of the venom proteomes of the Tunisian snakes *Cerastes cerastes*, *Cerastes vipera* and *Macrovipera lebetina* Proteomics 2005 5 16 4223 4235 10.1002/pmic.200402024 16206329

[B38] Retzios AD Markland FS Fibrinolytic enzymes from the venoms of *Agkistrodon contortrix contortrix* and *Crotalus basiliscus basiliscus*: cleavage site specificity towards the alpha-chain of fibrin Thromb Res 1994 74 4 355 367 10.1016/0049-3848(94)90151-1 8085237

[B39] Hahn BS Chang IM Kim YS Purification and characterization of piscivorase I and II, the fibrinolytic enzymes from eastern cottonmouth moccasin venom (*Agkistrodon piscivorus piscivorus*) Toxicon 1995 33 7 929 941 10.1016/0041-0101(95)00008-A 8588217

[B40] Siigur E Samel M Tõnismagi K Subbi J Siigur J Tu AT Biochemical characterization of lebetase, a direct-acting fibrinolytic enzyme from Vipera lebetina snake venom Thromb Res 1998 90 1 39 49 10.1016/S0049-3848(98)00009-7 9678676

[B41] Rodrigues VM Soares AM Guerra-Sá R Rodrigues V Fontes MR Giglio JR Structural and functional characterization of neuwiedase, a non-hemorrhagic fibrin(ogen)olytic metalloprotease from Bothrops neuwiedi snake venom Arch Biochem Biophys 2000 381 2 213 224 10.1006/abbi.2000.1958 11032408

[B42] Bello CA Hermogenes AL Magalhaes A Veiga SS Gremski LH Richardson M Isolation and biochemical characterization of a fibrinolytic proteinase from *Bothrops leucurus* (white-tailed Jararaca) snake venom Biochimie 2006 88 2 189 200 10.1016/j.biochi.2005.07.008 16139412

[B43] de Oliveira DG Murakami MT Cintra ACO Franco JJ Sampaio SV Arni RK Functional and structural analysis of two fibrinogen-activating enzymes isolated from the venoms of *Crotalus durissus terrificus* and *Crotalus durissus collilineatus* Acta Biochim Biophys Sin (Shanghai) 2009 41 1 21 29 10.1093/abbs/gmn003 19129947

[B44] Costa FL Rodrigues RS Izidoro LF Menaldo DL Hamaguchi A Homsi-Brandeburgo MI Biochemical and functional properties of a thrombin-like enzyme isolated from *Bothrops pauloensis* snake venom Toxicon 2009 54 6 725 735 10.1016/j.toxicon.2009.05.040 19539638

[B45] Zouari-Kessentini R Luis-José L Karray A Kallech-Ziri O Srairi-Abid N Bazaa A Two purified and characterized phospholipases A2 from *Cerastes cerastes* venom, that inhibits cancerous cell adhesion and migration Toxicon 2009 53 4 444 453 10.1016/j.toxicon.2009.01.003 19708222

[B46] Faure G Gowda VT Maroun RC Characterization of a human coagulation factor Xa-binding site on *Viperidae* snake venom phospholipases A_2_ by affinity binding studies and molecular bioinformatics BMC Struct Biol 2007 7 82 10.1186/1472-6807-7-82 18062812 PMC2248580

[B47] Franceschi A Rucavado A Mora N Gutiérrez JM Purification and characterization of BaH4, a haemorrhagic metalloproteinase from the venom of snake *Bothrops asper* Toxicon 2000 38 1 63 77 10.1016/S0041-0101(99)00127-0 10669012

[B48] Marrakchi N Barbouche R Guermazi S Bon C el Ayeb M Procoagulant and platelet-aggregating properties of Cerastocytin from Cerastes cerastes venom Toxicon 1997 35 2 261 272 10.1016/S0041-0101(96)00116-X 9080583

